# Trends in socioeconomic inequalities in child malnutrition in Vietnam: findings from the Multiple Indicator Cluster Surveys, 2000–2011

**DOI:** 10.3402/gha.v9.29263

**Published:** 2016-02-29

**Authors:** Vu Duy Kien, Hwa-Young Lee, You-Seon Nam, Juhwan Oh, Kim Bao Giang, Hoang Van Minh

**Affiliations:** 1 Center for Population Health Sciences, Hanoi School of Public Health, Hanoi, Vietnam; 2 Center for Health System Research, Hanoi Medical University, Hanoi, Vietnam; 3 Unit of Epidemiology and Global Health, Department of Public Health and Clinical Medicine, Umeå University, Umeå, Sweden; 4 JW Lee Center for Global Medicine, Seoul National University College of Medicine, Seoul, Korea; 5 Department of Family Medicine, Seoul National University Hospital, Seoul, Korea; 6 Institute for Preventive Medicine and Public Health, Hanoi Medical University, Hanoi, Vietnam

**Keywords:** trend, malnutrition, inequality, decomposition, Vietnam

## Abstract

**Background:**

Child malnutrition is not only a major contributor to child mortality and morbidity, but it can also determine socioeconomic status in adult life. The rate of under-five child malnutrition in Vietnam has significantly decreased, but associated inequality issues still need attention.

**Objective:**

This study aims to explore trends, contributing factors, and changes in inequalities for under-five child malnutrition in Vietnam between 2000 and 2011.

**Design:**

Data were drawn from the Viet Nam Multiple Indicator Cluster Survey for the years 2000 and 2011. The dependent variables used for the study were stunting, underweight, and wasting of under-five children. The concentration index was calculated to see the magnitude of child malnutrition, and the inequality was decomposed to understand the contributions of determinants to child malnutrition. The total differential decomposition was used to identify and explore factors contributing to changes in child malnutrition inequalities.

**Results:**

Inequality in child malnutrition increased between 2000 and 2011, even though the overall rate declined. Most of the inequality in malnutrition was due to ethnicity and socioeconomic status. The total differential decomposition showed that the biggest and second biggest contributors to the changes in underweight inequalities were age and socioeconomic status, respectively. Socioeconomic status was the largest contributor to inequalities in stunting.

**Conclusions:**

Although the overall level of child malnutrition was improved in Vietnam, there were significant differences in under-five child malnutrition that favored those who were more advantaged in socioeconomic terms. The impact of socioeconomic inequalities in child malnutrition has increased over time. Multifaceted approaches, connecting several relevant ministries and sectors, may be necessary to reduce inequalities in childhood malnutrition.

## Introduction

Child malnutrition affects children's physical and cognitive development, diminishes immunity, and impacts child mortality and morbidity (1, 2). It is not surprising therefore that malnutrition is, by far, one of the largest contributors to the global burden of disease (3). In addition, malnutrition in early childhood is associated with functional impairment in adulthood. For example, childhood malnutrition can lower work capacity and economic productivity (4) and affect socioeconomic status in later life. As with many other health indicators, malnutrition displays large inequalities across different socioeconomic groups in most developing countries (5).

Vietnam achieved dramatic economic development during a relatively brief time span since the economic reform (*Doi Moi*) and accordingly has shown big improvements in various health indicators. Child malnutrition in Vietnam has improved in general over the last couple of decades, from 41% in 1990 to 15.3% in 2013 (6). However, Vietnam still ranks as having one of the highest child malnutrition rates among Asian countries (7). In addition, the benefits from economic improvement have not been distributed uniformly, with a large share going to small groups of economically advantaged people (8). This situation has worsened inequalities in health status, with child malnutrition being one of the most unequal health indicators, in spite of the decline in the overall proportion of malnutrition (9).

A few studies in developing countries have explored socioeconomic inequalities in malnutrition and associated factors using various proxy indicators for socioeconomic status and analytical methods. However, most studies employed stunting, underweight, or (possibly) wasting as forms of malnutrition indices. Hong (10) investigated inequality in stunting according to household socioeconomic status (measured by household ownership of durable assets) through bivariate and multivariable logistic regression using the 2003 Ghana Demographic and Health Survey data. Their study showed a strong association between low socioeconomic status and high probability of stunting. Van de Poel et al. (11) reported on socioeconomic inequality in child malnutrition using a concentration index (CI) drawn from data in 47 developing countries. The authors argued that socioeconomic inequality in child malnutrition existed throughout the developing countries and was not related to the average malnutrition rate. Mazumdar (12) measured and decomposed India's inequality in childhood stunting in 2005 using an asset-based measure of socioeconomic status for calculating the CI; he showed that the biggest contributor to inequality in stunting was household socioeconomic status.

In Vietnam, some studies show that child nutrition is affected by environmental, socioeconomic, and political factors (9, 13). In addition, much is known about the national trends in Vietnamese child malnutrition (14, 15). However there is little information on how socioeconomic inequalities in malnutrition have evolved over time or what determinants are major contributors to changing inequalities in child malnutrition. Thang and Popkin (16) identified factors that may affect high rates of stunting and underweight in Vietnamese children by multivariable logistic regression. Thang and Popkin (9) further studied changes in inequality for child malnutrition in Vietnam from 1992–1993 to 1997–1998. However, both of the studies excluded children under 2 years of age from the analysis. Wagstaff et al. (17) calculated the CI of malnutrition for Vietnamese children under 10 using the 1993 and 1998 data. They also reported results of a decomposition of inequality and the changing inequality of malnutrition through the differential decomposition method. However, this study provides insufficient information for targeting the fourth Millennium Development Goal (MDG), which concerns reducing the mortality rate of children under five, because it included children under the age of 10 (18). In addition the data from Wagstaff's study are now out of date.

This study aims to provide updated information about inequality in Vietnamese child malnutrition, such as stunting, underweight, and wasting, since 2000, focusing only on children under age five. Specifically, two main points will be addressed in this study: 1) inequality in child malnutrition according to socioeconomic status in 2000 and 2011 and factors that contributed to the inequality in each year; 2) changes in inequalities in child malnutrition according to socioeconomic status between 2000 and 2011 and factors that contributed to the change in inequality across these years.

## Methods

### Study data

We analyzed data from two rounds of the Multiple Indicator Cluster Survey (MICS), specifically from 2000 to 2011 in Vietnam, to examine the trends in socioeconomic inequalities for under-five child malnutrition (19, 20). There was another round in 2006 between the two rounds of MICS used in the analysis. However, data from that round are not included in our analysis because malnutrition information was not available in the data set for that year (21). The MICS was designed by the United Nations Children's Fund (UNICEF) with the purpose of collecting internationally comparable data for women and children. In Vietnam, the MICS were carried out by the General Statistics Office of Vietnam with financial and technical support from UNICEF and the United Nations Population Fund. The sample was a two-stage, probability sample, stratified and clustered (20, 22). In 2000, the MICS included complete information for 3,104 under-five children, with a response rate of 99.9% (22), while in 2011, the MICS included a sample of 3,678 under-five children, with a response rate of 98.6% (20). Details of these MICS have been described elsewhere (20, 22). For this study, data from the 2000 and 2011 MICS were accessed and analyzed with the authorization of UNICEF.

### Outcome and explanatory variables

The outcome variable in this study was under-five child malnutrition categorized into underweight, stunting, and wasting. In the MICS data, these outcomes were measured and converted to z-scores. Since reference z-values measured by the US National Center for Health Statistics (NCHS) were available in both the 2000 and 2011 data, while the new WHO z-score standard values introduced in April 2006 were not available in the 2000 MICS (20), we used the NCHS reference in order to compare the malnutrition status between 2000 and 2011 by the same unit. The z-scores were estimated for different variables of under-five child weight for age, height for age and weight for height (20, 22). Following WHO guidelines, under-five children with a z-score of less than two on each of these variables were classified as underweight, stunted, and wasted, respectively. Under-five children who are stunted and wasted suggest chronic and acute undernutrition, respectively, whereas underweight under-five children are used as a composite indicator to reflect both acute and chronic malnutrition (23). For calculations of the percentages of child malnutrition and the logistic regression model, we converted these outcome variables into binary variables.

The explanatory variables of interest in this study were as follows: child's age (months), child's sex, living area (urban/rural), ethnicity (minority, Kinh, or Hoa), mother's education level, and household socioeconomic status, all of which have been shown to be important determinants for child malnutrition (9, 16) (24) and were available in our data set (20, 22). Safe water and sanitation were not included in the model because these variables were used to construct the wealth asset index (20, 22).

### Measurement of socioeconomic status

In this study, we used the wealth asset index as a proxy for socioeconomic status. The wealth asset index was constructed by principal components analysis using information on the ownership of consumer goods, dwelling characteristics, water and sanitation, and other characteristics that are related to household wealth in both the 2000 and 2011 MICS data sets (20, 22). The assets and other characteristics related to wealth used in these calculations were as follows: water sources, toilet facility, housing, fuel types for cooking, electricity, bank account, durable goods (such as a radio, TV, refrigerator, fixed telephone, watch, mobile phone, bicycle, motorcycle, motorized boat, car), and animals (such as buffalo, cattle, horses, donkeys, goats, sheep, chickens, pigs). The wealth scores were divided into five socioeconomic status quintiles (from the poorest to the richest) after being estimated and assigned for each household. The method for estimating wealth asset index has been described in detail elsewhere (20, 22).

### Health inequality analysis

To measure the degree of socioeconomic inequality in under-five child underweight, stunting, and wasting, we used the CI (25, 26). The CI is calculated as twice the area between the concentration curve and the line of equality (the 45-degree line). O'Donnell (26) described the formula for CI as follows:

1 $$\text{C}=\frac{2}{\mu }\mathrm{cov}(h,r)$$

Here, µ is the mean of under-five child malnutrition (underweight, stunting, or wasting), *h* represents the values of under-five child malnutrition (underweight, stunting, or wasting) of each observation, and *r* is the rank of the household socioeconomic status. The CIs of under-five child underweight, stunting, and wasting could range between −1 and +1. The CI takes a value of 0, if the distribution of under-five child underweight, stunting, and wasting prevalence is completely equal between the rich and the poor. If it is negative, it indicates that the concentration of under-five malnutrition is higher among the poor, and if it is positive it indicates that the concentration of under-five malnutrition is higher among the rich (26). To increase the precision of the estimation, we used the continuous variables of under-five child underweight, stunting, and wasting to estimate the CIs. In addition, the household socioeconomic status was also used in the continuous form.

### Decomposition of socioeconomic inequalities and their change

In this study, we decomposed the CIs of under-five child underweight and stunting in 2000 and 2011. Decomposition of the CIs helps in understanding the contribution of the determinant variables to socioeconomic inequalities in the health outcome variable (17, 26). For a continuous outcome variable, a linear regression model linking the outcome variable (*y*) to the set of *k* determinants (*x*_*k*_) can be represented as follows:

2 $$y=\alpha +\sum_{k}\beta_{k}x_{k}+\varepsilon$$

where *β*_*k*_ is the coefficient of *x*_*k*_ and *ɛ* is the error term. Equation 2 can be transformed to the CI for *y*, and it can be written as follows:

3 $$C=\sum_{k}\left(\beta_{k}{\bar{x}}_{k}/\mu \right)C_{k}+GC_{\in }/\mu$$

where µ is the mean of *y* (the outcome variable); ${\bar{\text{x}}}_{\text{k}}$ is the mean of x_k_ (the *k*th determinant variable); C_k_ is the CI for x_k_, and GC_∈_ is the generalized concentration for the error term (ɛ). The element $\left(\beta_{k}{\bar{x}}_{k}/\mu \right)C_{k}$ is an explained component, while the element GC_∈_/μ is an unexplained component (or residual). In the explained component, $\left(\beta_{k}{\bar{x}}_{k}/\mu \right)$ is elasticity that indicates the impact of each C_k_ on the total CI of *y* (17, 26).

In addition, the decomposition of the changes of CIs was also applied to identify the contribution of different determinants to those changes. We decomposed the changes in CIs of underweight and stunted under-five children in 2000 and 2011. To decompose the changes in CIs, we applied the total differential decomposition, which enables estimation of the overall impacts on under-five children's malnutrition inequalities on changes in regression coefficients, changes in the means of the determinants of malnutrition, and changes in the degree of inequality in the determinants of malnutrition. The equation for this decomposition was proposed by Wagstaff et al. (17), as follows:

4 $$dc=-\frac{c}{\mu }d\alpha +\sum_{k}\frac{{\bar{x}}_{k}}{\mu }(C_{k}-C)d\beta_{k}+\sum_{k}\frac{\beta_{k}}{\mu }(C_{k}-C)d{\bar{x}}_{k}+\sum_{k}\frac{\beta_{k}{\bar{x}}_{k}}{\mu }dC_{k}+d\frac{GC_{\varepsilon }}{\mu }.$$

where *dC*, *dα*, *dβ*_*k*_, $d{\bar{x}}_{k}$, *dC*_*k*_, and $d\frac{GC_{\varepsilon }}{\mu }$ are changes in the total CI, constant value, coefficients of each determinant, means of each determinants, CI of each determinant, and error value, respectively. The estimation method for total decomposition of changes has been described in detail elsewhere (17).

### Statistical methods

All statistical analyses were carried out using Stata^®^ 13.1. Proportions were compared by using the chi-square test, and the z-test was used to assess statistically significant differences between the two proportions. The Distributive Analysis Stata Package (DASP) (27) with the command **igini** was used to calculate the CIs of under-five child underweight, stunting, and wasting. The command **digini** provided the results of the statistical test if the CIs were statistically significantly different from 0, and the difference between the CIs of two different sets of the study population (using independence two-tailed t-test). Multivariable analysis was conducted with logistic regression for the binary outcome variables underweight, stunting, and wasting. All analysis used the survey-related commands in STATA, with weighting factors for children from the data set (20, 22). The level of statistical significance was set to 0.05.

## Results

### Trends in child malnutrition and socioeconomic inequality

Table 1 shows an overview of the distribution of under-five child malnutrition by socioeconomic status and the difference between 2000 and 2011. The overall prevalence of child underweight, stunting, and wasting significantly declined during 2000 and 2011 (the absolute reduction was 21.4, 14, and 1.7%, respectively), although the absolute reduction of wasting was not as remarkable. A more detailed look revealed that significant reductions occurred in almost all of the socioeconomic status groups in underweight and stunting, while only the richest group showed significant reduction in wasting. As shown in Table 2, the CIs of underweight, stunting, and wasting were significantly different from 0 and showed negative values, indicating that poor children had a higher possibility of being underweight, stunted, or wasted. Moreover, all absolute values of the CI of underweight and stunting in 2011 were greater than that in 2000, indicating that under-five children's underweight and stunting inequalities increased throughout this period. The difference in the CI for wasting between 2000 and 2011 was not significant (*p*>0.05) but differences in the CI for underweight and stunting were significant (*p*<0.001).

**Table 1:** Percentage of under-five child malnutrition by socioeconomic status, 2000 and 2011

	Prevalence of malnutrition by socioeconomic status, % (SE)					
	
	Poorest	Poorer	Middle	Richer	Richest	All
Underweight (weight for age <2 SD)						
Year 2000	42.3 (2.2)	36.1 (2.6)	31.1 (3.8)	29.4 (2.2)	17.6 (2.6)	33.1 (1.7)
Year 2011	20.6 (1.6)	11.3 (1.2)	14.0 (1.4)	8.4 (1.3)	3.0 (0.6)	11.7 (0.6)
Diff-1	21.7 (3.1)^a^	24.8 (2.6)^a^	17.1 (3.6)^a^	21.0 (2.3)^a^	14.6 (2.7)^a^	21.4 (1.7)^a^
Stunted (height for age <2 SD)						
Year 2000	45.8 (3.3)	42.5 (3.9)	37.2 (3.4)	31.5 (1.8)	16.6 (2.8)	36.7 (2.4)
Year 2011	40.8 (1.5)	24.2 (2.3)	24.3 (1.9)	15.7 (1.4)	6.1 (0.8)	22.7 (1.1)
Diff-2	4.9 (3.6)	18.3 (5.1)^a^	12.9 (3.4)^a^	15.8 (2.1)^a^	10.6 (3.1)^a^	14.0 (2.4)^a^
Wasted (weight for height <2 SD)						
Year 2000	7.7 (1.3)	4.9 (1.1)	4.3 (1.1)	5.2 (1.0)	5.1 (1.1)	5.7 (0.6)
Year 2011	5.2 (0.7)	4.1 (0.7)	4.5 (0.8)	4.2 (0.9)	1.8 (0.4)	4.0 (0.4)
Diff-3	2.6 (1.6)	0.7 (1.3)	−0.2 (1.4)	1.0 (1.3)	3.4 (1.1)^b^	1.7 (0.7)^c^

Diff-1, Diff-2, Diff-3: the difference between 2000 and 2011 of percentage of under-five children who were underweight, stunted, and wasted, respectively. SD: standard deviation; SE: standard error.

a,b,c Significant at 0.05, 0.01, and 0.001, respectively (using a chi-square test).

**Table 2:** Concentration indices (CIs) of under-five child malnutrition, 2000 and 2011

	Underweight (weight for age <2 SD)		Stunted (height for age <2 SD)		Wasted (weight for height <2 SD)	
			
	CI (SE)	p*	CI (SE)	p*	CI (SE)	p*
Year 2000	−0.15 (0.02)	<0.001	−0.17 (0.03)	<0.001	−0.09 (0.05)	0.12
Year 2011	−0.29 (0.03)	<0.001	−0.32 (0.02)	<0.001	−0.13 (0.05)	0.01
Diff	−0.14 (0.04)	<0.001	−0.15 (0.03)	<0.001	−0.04 (0.07)	0.51

CI: concentration index; SE: standard error. Diff: the difference in CI of under-five child malnutrition between 2000 and 2011.

* Independence two-tailed *t*-test to compare the values with 0.

### Determinants of child malnutrition

Table 3 presents the determinants associated with under-five child malnutrition. The results in Table 3 once again confirm the results in Table 1, by revealing that even after adjusting for other determinants the reduction in underweight, stunting, and wasting among under-five children was still significant (all *p*<0.05). The main factors that showed significant association with under-five child underweight were being older, belonging to a minority, having a mother with lower education, and belonging to a lower socioeconomic status group. The same factors were significantly associated with under-five child stunting and underweight, with the exception of living in rural areas, which was significantly associated with stunting. Factors significantly associated with under-five child wasting were living area and socioeconomic status.

**Table 3:** Determinants of under-five child malnutrition, 2000 and 2011: multivariable logistic regression analysis

	Underweight (weight for age <2 SD)		Stunted (height for age <2 SD)		Wasted (weight for height <2 SD)	
			
	OR (95% CI)	*p*	OR (95% CI)	*p*	OR (95% CI)	*p*
Year (2011 vs. 2000)	0.89 (0.88–0.91)	<0.001	0.95 (0.93–0.97)	<0.001	0.97 (0.94–0.99)	0.032
Child's age (months)	1.10 (1.08–1.12)	<0.001	1.09 (1.07–1.11)	<0.001	0.98 (0.95–1.01)	0.182
Child's age (squares)	0.99 (0.98–0.99)	<0.001	0.99 (0.98–0.99)	<0.001	0.99 (0.98–0.99)	0.228
Sex of child						
Female	1.14 (0.98–1.31)	0.085	1.04 (0.92–1.18)	0.494	0.83 (0.64–1.09)	0.188
Male	1		1		1	
Area						
Rural	1.16 (0.90–1.49)	0.257	1.34 (1.09–1.65)	0.007	0.65 (0.47–0.89)	0.008
Urban	1		1		1	
Ethnicity						
Minorities	1.56 (1.25–1.95)	<0.001	1.71 (1.44–2.03)	<0.001	1.14 (0.68–1.91)	0.627
Kinh/Hoa	1		1		1	
Mother's education						
Primary or less	1.29 (1.02–1.62)	0.036	1.39 (1.05–1.85)	0.023	1.33 (0.86–2.05)	0.201
Lower secondary	1.14 (0.9–1.44)	0.292	1.25 (1–1.57)	0.048	1.2 (0.78–1.84)	0.398
Upper secondary and tertiary	1		1		1	
Socioeconomic status						
1st quintile (poorest)	2.94 (2.23–3.88)	<0.001	3.46 (2.51–4.77)	<0.001	2.23 (1.33–3.76)	0.003
2nd quintile	2.33 (1.66–3.27)	<0.001	2.84 (2.07–3.89)	<0.001	1.62 (1.01–2.59)	0.045
3rd quintile	2.45 (1.69–3.56)	<0.001	2.80 (2.02–3.88)	<0.001	1.63 (1.01–2.63)	0.045
4th quintile	1.95 (1.41–2.69)	<0.001	2.01 (1.47–2.76)	<0.001	1.64 (1.09–2.46)	0.017
5th quintile (richest)	1		1		1	

OR: odds ratio; SD: standard deviation. Underweight, stunted, and wasted were converted to binary variables (if the value <2 SD=1, otherwise=0).

### Decomposition of socioeconomic inequality in child malnutrition

Table 4 shows absolute and relative estimates of the contribution to overall CIs of under-five child underweight and stunting. We did not decompose the CI of wasting because there was no significant difference between 2000 and 2011 (Table 2). Almost every determinant made a positive contribution to lowering the CIs, meaning that the relevant determinant increased the inequality. As shown in Table 4, the major determinants contributing to socioeconomic inequalities in under-five child underweight in both 2000 and 2011 were children's ethnicity and socioeconomic status. More than one-half of socioeconomic inequality reflects the direct contribution of socioeconomic status, while the remainder is influenced by other factors. The contribution of mother's level of education was 25% in 2000 but negligible in 2011.

**Table 4:** Decomposition of concentration indices for under-five child underweight and stunted, 2000 and 2011

	Underweight (weight for age <2 SD)				Stunting (height for age <2 SD)			
		
	Year 2000		Year 2011		Year 2000		Year 2011	
				
	Contribution^b^	%	Contribution^b^	%	Contribution^b^	%	Contribution^b^	%
Child's age (months)	−0.003	3.2	−0.005	1.5	−0.002	2.1	−0.015	0.0
Child's age (squares)	−0.002		0.001		−0.002		0.015	
Sex of child (female vs. male)	0.000	−0.2	0.000	0.1	0.000	−0.2	−0.001	0.2
Area (rural vs. urban)	0.003	−1.9	−0.024	8.2	−0.015	9.0	−0.022	6.8
Ethnicity (minority vs. Kinh/Hoa)	−0.038	24.7	−0.053	18.1	−0.062	36.4	−0.040	12.5
Mother's education		25.7		0.1		24.8		3.7
Primary or less	−0.043		0.001		−0.046		−0.009	
Lower secondary	0.004		−0.001		0.004		−0.002	
Upper secondary and tertiary (ref.)								
Socioeconomic status		50.9		66.9		28.2		72.0
1st quintile (poorest)	−0.101		−0.195		−0.067		−0.216	
2nd quintile	−0.016		−0.034		−0.017		−0.037	
3rd quintile	0.011		0.002		0.012		0.002	
4th quintile	0.028		0.029		0.024		0.024	
5th quintile (richest) (ref.)								
Residual	0.004	−2.4	−0.015	5.1	0.001	−0.3	−0.015	4.8
Total^a^	−0.15	100	−0.29	100	−0.17	100	−0.32	100

SD: standard deviation.

a Total concentration indices

b contributions to the concentration indices.

The determinants that made the biggest contribution to socioeconomic inequalities in under-five child stunting in 2000 and 2011 also showed the same pattern. Specifically, ethnicity, socioeconomic status, and mother's education level were the biggest contributors in 2000, although the influence of mother's education level on inequality in malnutrition disappeared in 2011. The contribution of socioeconomic status increased during the period 2000–2011 (from 50.9 to 66.9% for under-five child underweight and from 28.2 to 72.0% for under-five child stunting). In 2011, all variables contributed to widening socioeconomic status in malnutrition. The contributions of residuals were relatively low, suggesting that the decomposition model explained the socioeconomic inequality in malnutrition quite well.

### Decomposition of change in socioeconomic inequality in child malnutrition

The contributions to the changes in the inequalities are presented as percentages, with positive percentages indicating a contribution to an increase in inequality and negative percentages indicating a contribution to a decrease in inequality. Table 5 shows the decomposition of changes in CIs during the period 2000–2011. The biggest contributor to the change in underweight inequalities between 2000 and 2011 was age (73%), and the second biggest contributor was socioeconomic status (15.5%). However the biggest influence on the change in stunting inequality was by far socioeconomic status (63.5%). In addition, unexplained factors also contributed to increasing inequalities in both underweight and stunting between 2000 and 2011 (16.3 and 18.7%, respectively). By contrast, belonging to an ethnic minority decreased inequalities between 2000 and 2011 in under-five child underweight (−9.1% and stunting −17.7%).

**Table 5:** Decomposition of change in concentration indices for under-five child underweight and stunted, 2000 and 2011: total differential decomposition

	Change in CI of underweight between 2000 and 2011					Change in CI of stunting between 2000 and 2011				
		
	*B* a	Means of *x* b	CI^c^	Total^d^	%	*B* a	Means of *x* b	CI^c^	Total^d^	%
Child's age (months)	−0.282	−0.022	−0.017	−0.321	73.0	−0.085	−0.018	−0.012	−0.115	22.0
Child's age squares	0.173	0.020	0.023	0.217		0.050	0.017	0.017	0.083	
Sex of child (female vs. male)	−0.011	−0.000	0.001	−0.011	7.4	−0.013	−0.000	0.001	−0.012	8.4
Area (rural vs. urban)	0.000	0.000	0.001	0.001	−0.6	−0.001	−0.000	−0.004	−0.004	3.0
Ethnicity (minorities vs. Kinh/Hoa)	0.013	0.006	−0.006	0.013	−9.1	0.026	0.010	−0.009	0.026	−17.7
Mother's education	0.014	0.009	−0.019	0.004	−2.5	0.009	0.008	−0.021	−0.003	2.1
Primary or less	0.024	0.009	−0.012	0.021		0.019	0.009	−0.013	0.015	
Lower secondary	−0.010	−0.000	−0.007	−0.017		−0.010	−0.001	−0.008	−0.018	
Upper secondary and tertiary (ref.)										
Socioeconomic status	−0.011	0.033	−0.044	−0.022	15.5	−0.078	0.026	−0.04	−0.093	63.5
1st quintile (poorest)	0.015	0.020	−0.011	0.024		−0.064	0.013	−0.007	−0.058	
2nd quintile	0.002	0.000	−0.016	−0.014		0.000	0.000	−0.017	−0.016	
3rd quintile	−0.005	0.003	−0.011	−0.012		−0.000	0.004	−0.011	−0.008	
4th quintile	−0.023	0.010	−0.006	−0.020		−0.014	0.009	−0.005	−0.011	
5th quintile (richest) (ref.)										
Residual (unexplained part)	0.000	0.000	0.000	−0.023	16.3	0.000	0.000	0.000	−0.027	18.7
Total	−0.105	0.047	−0.061	−0.142	100	−0.092	0.042	−0.069	−0.147	100

SD: standard deviation.

a,b,c Contributions by coefficient, mean, concentration index of determinants, respectively

d contributions to the change in concentration indices.

The total differential decomposition on socioeconomic inequality in underweight and stunting in Table 5 shows that the contribution of the increased regression coefficient and increased inequality of socioeconomic status reinforced one another by widening the inequality of underweight and stunting, and the mean level of socioeconomic status offset those effects. The CI value changed by approximately −0.142 and −0.147 in underweight and stunting, respectively, whereas CI changes that occurred through changes in the degree of inequality in the determinants of malnutrition were −0.061 and −0.069, suggesting that there is more to rising inequality in malnutrition than rising inequalities in their determinants.

## Discussion

Although the results of this study are drawn from cross-sectional data and therefore causality cannot be claimed, our paper lays the groundwork for unraveling the causes and changes of inequalities in malnutrition for Vietnamese children under five and highlights the links between socioeconomic inequalities and malnutrition. The findings of this research carry a few important policy implications.

First of all, socioeconomic inequalities in Vietnamese under-five child malnutrition have significantly increased since 2000, although the overall prevalence of malnutrition itself declined. As mentioned, Vietnam experienced rapid economic growth after its dramatic economic reforms (*Doi Moi*), and there were also major improvements in health status. This was not only the result of changes in the health sector but was also due to a combination of changes in income, lifestyle, and other factors (28). However, the benefits of this development favored the more prosperous groups at the expense of those less prosperous (8). Consequently, inequality in overall health status, as well as malnutrition in under-five children, has worsened, while average health indicators have improved.

Secondly, another finding from the decomposition of the CI was that most of the determinants made a positive contribution to the socioeconomic inequality in malnutrition. This means that the combined result of the marginal effect of each determinant on malnutrition and its distribution by socioeconomic status was to raise socioeconomic inequality in child malnutrition, with malnutrition being more prevalent among the poor. The result may occur either because particular determinants associated with higher malnutrition were more common among people with lower socioeconomic status or because some determinants associated with a lower malnutrition risk were more prevalent among people with higher socioeconomic status.

The contribution presented as percentages of overall socioeconomic inequality clearly shows that most of the inequality in Vietnamese child malnutrition in both 2000 and 2011 was attributable to socioeconomic status and ethnicity. Socioeconomic status accounted for almost two-thirds of the total inequality in both underweight and stunting in 2000 and 2011, meaning that about 50.9 to 72.0% of socioeconomic status–related inequality in malnutrition can be explained by socioeconomic status itself. Ethnicity was ranked as the second contributor in both 2000 and 2011 even though the contribution declined significantly. Although Vietnam is a multi-ethnic country, the main ethnic group in Vietnam is Kinh, which comprises over 85% of the total population. Inferiority in the socioeconomic status of minorities has been a long-lasting problem in Vietnam. The ethnic minorities mostly live in mountainous or remote areas, where economic or cultural benefit cannot be reached easily. This study confirmed again that poor children are more likely to belong to minorities, who are consequently more likely to experience malnutrition. This result is consistent with Wagstaff's study, which proved that inequalities in household consumption and commune fixed effects were the biggest contributors to inequality in malnutrition (17). Even though the two studies cannot be directly compared, because our study did not include variables for the commune effect and Wagstaff's did not include ethnicity, what is clear is that socioeconomic status has been an incorrigible cause of inequality in child malnutrition since 1990.

On the other hand, the contribution of mother's education sharply declined between 2000 and 2011. This result might be because those economically disadvantaged women were given more opportunities to get a better education, as the national economic levels increased and the position of women in society was elevated. Thus, upgrading mothers’ education might not be a policy priority for tackling child malnutrition.

The results of the decomposition of changes in the CI of malnutrition showing how much of a contribution each determinant made to worsening the inequality in malnutrition between 2000 and 2011 yielded another key finding, that is, that the reasons for the changes in inequality for underweight and stunting between 2000 and 2011 were completely different. Socioeconomic status itself accounted for a big portion of the total change in the socioeconomic inequality of stunting, although it did not make a big contribution to underweight. A supporting reason for this can be thought as follows: underweight can be relatively easily corrected just by providing appropriate feeding, which does not demand difficult measures; however, stunting is an indicator of a more chronic malnutrition status (29). It cannot be normalized in a short period of time by simple measures, but needs more comprehensive environmental support, relying more on socioeconomic status. In light of this fact, our results can be interpreted in the context that parents in lower economic levels became capable of feeding their children enough so that they did not become underweight, but they still could not give enough comprehensive care to prevent stunting. Age of the child was the biggest contributor to worsening inequalities in malnutrition. Under-five children who were underweight were more likely to be relatively older and living in poorer households.

Table 5 provides insights into what components of socioeconomic status led to the rise in inequality in malnutrition. In cases of both underweight and stunting, rising inequality and the marginal effect of socioeconomic status on malnutrition were the main drivers for the rising inequality in child malnutrition, while the overall improvement of socioeconomic status offset worsening inequality. Given the fact that the fees for both public and private health-care services have been rising significantly and that expensive high quality foods now have greater availability, the increased marginal impact of socioeconomic status on nutritional status is plausible (30).

The results of the main determinants for the average level of malnutrition (Table 3) and the causes of changing inequality (Table 5) imply that there is a possible trade-off between reducing the improving mean level of the variable and relative inequality. For example, higher socioeconomic status reduced the odds of childhood stunting (Table 3), but an increase in the relative inequality in stunting was caused partly by a growing inequality in socioeconomic status and partly by a lowering in average socioeconomic status (Table 5).

This study has a caveat that commonly occurs in cross-sectional studies. The results must be interpreted with caution so that they are not interpreted as implying causality. In addition, unknown factors that were not included in our analysis also made a non-negligible contribution by worsening inequality in both underweight and stunting. This suggests a need for further studies to identify and tackle those factors. Despite these weaknesses, the findings from this study have some meaningful policy implications. First, like other studies, this study confirms that there is some trade-off between improvement in averages and improvement in distributions. Policy measures relying only on the country's average values can be misleading. In addition, this study drilled down to identify factors that contributed to the recognized inequalities and changes in inequalities over time, thereby enabling us to better judge where we have to focus in the future in order to tackle inequalities in child malnutrition.

## Conclusions

In order to address inequalities in child malnutrition in Vietnam, special attention should be given to the policy measures that narrow socioeconomic gaps between groups in the population. Our study confirms that most inequalities in Vietnamese children's malnutrition resulted from socioeconomic inequalities. To remedy this problem, the Vietnamese government needs to direct efforts towards raising socioeconomic status in minorities and focusing on older children. Specifically, investment in education, empowerment of economically disadvantaged groups, and creation of greater working opportunities would be important measures. There is a need for a comprehensive approach beyond the health sector with a sharing of efforts between other ministries.
